# Antibodies towards Tyrosine Amyloid-Like Fibrils Allow Toxicity Modulation and Cellular Imaging of the Assemblies

**DOI:** 10.3390/molecules23061273

**Published:** 2018-05-26

**Authors:** Dor Zaguri, Topaz Kreiser, Shira Shaham-Niv, Ehud Gazit

**Affiliations:** 1 Department of Molecular Microbiology and Biotechnology, George S. Wise Faculty of Life Sciences, Tel Aviv University, Tel Aviv 6997801, Israel; dorzaguri@mail.tau.ac.il (D.Z.); topazk136@gmail.com (T.K.); shira.shaham@gmail.com (S.S.-N.); 2 Department of Materials Science and Engineering, Iby and Aladar Fleischman Faculty of Engineering, Tel Aviv University, Tel Aviv 6997801, Israel; 3 BLAVATNIK CENTER for Drug Discovery, Tel Aviv University, Tel Aviv 6997801, Israel

**Keywords:** self-assembly, tyrosine, metabolite amyloid-like fibrils, tyrosinemia, inborn error of metabolism, immunogenicity, anti-tyr antibodies

## Abstract

The amino acid tyrosine forms cytotoxic amyloid-like fibrils by molecular self-assembly. However, the production of antibodies towards tyrosine assemblies, reflecting their presentation to the immune system, was not demonstrated yet. Here, we describe the production of antibodies that specifically recognize tyrosine in its fibrillated form. The antibodies were demonstrated to specifically bind self-assembled tyrosine, in contrast to its non-aggregated form or disintegrated fibrils. The antibodies could be used for immunostaining of tyrosine fibrils in cultured cells. Furthermore, confocal microscopy allowed a demonstration of the intracellular presence of the metabolite amyloids in a neuroblastoma cell model. Finally, pre-incubation of tyrosine fibrils with the antibodies resulted in significant reduction in their cytotoxicity. Taken together, we provide an experimental proof for the immunogenicity of tyrosine amyloid fibrillary assemblies. These specific antibodies against tyrosine structures could be further used as a research tool to study the dynamics, toxicity and cellular localization of the assemblies.

## 1. Introduction

The self-assembly of proteins and polypeptides into amyloid fibrils is a major hallmark of various degenerative diseases, including Alzheimer’s disease, Parkinson’s disease and Type 2 diabetes [1,2,3]. Although formed by a structurally diverse group of proteins, all amyloid fibrils share similar biophysical and structural properties [4,5,6]. Over the last two decades, a reductionist approach using increasingly shorter amyloid peptide fragments, including minimal dipeptides, has demonstrated the formation of typical amyloid fibrils. These fibrils shows the same biophysical and structural features characteristic of the assemblies formed by full length proteins and polypeptides [5,7,8,9]. Moreover, it has been recently demonstrated that several metabolites, including both single amino acids and nucleic bases, can form well-ordered amyloid-like fibrillar assemblies. These fibrils were shown to bind the amyloid-specific, dyes Thioflavin T (ThT) and Congo red, and to trigger cytotoxicity by inducing apoptotic cell death as observed for protein and polypeptide amyloid structures [10,11,12,13,14]. These discoveries thus extended the metabolite amyloids hypothesis, suggesting that small, monomeric metabolites can self-assemble to form amyloid-like fibrils showing similar properties to proteinaceous amyloids.

These metabolites that form amyloid-like assemblies, were known to accumulate pathologically in various inborn error of metabolism (IEM) disorders. This family of disorders results from mutations in single genes, leading to cellular failure to perform critical metabolic reactions. In most of these disorders, the pathological manifestation results from the accumulation of metabolites, which hinder the normal function of cells and tissues. Unless these inborn genetic disorders are treated with a strict diet, they may result in mental retardation and other developmental abnormalities. Although most of the reported IEM conditions are rare, as a group, IEM disorders comprise a very considerable portion of pediatric genetic diseases [15,16,17]. Since the molecular basis of tissue damage is poorly understood, no disease-modifying treatment is available.

Specifically, the aromatic l-Tyrosine (Tyr) amino acid was found to form amyloid-like fibrils [13,18]. The accumulation of Tyr occurs in three different types of tyrosinemia, all resulting from autosomal recessive mutations in several genes in the phenylalanine (Phe) and Tyr metabolic pathway [19]. Type I tyrosinemia results from a mutation in the *FAH* gene, which encodes the fumarylacetoacetate hydrolase enzyme. The mutation leads to fumarylacetoacetate accumulation, which inhibits previous steps in the tyrosine degradation pathway, resulting in accumulation of tyrosine in proximal renal tubular cells and in hepatocytes leading to kidney and liver damage respectively [20]. Type II tyrosinemia results from a mutation in the *TAT* gene, which encodes the tyrosine aminotransferase enzyme. As a result of TAT enzyme deficiency, tyrosine accumulates, causing ophthalmologic and dermatologic abnormalities [21]. The rarest of the three conditions, type III tyrosinemia, results from a mutation in the *HPD* gene, which encodes the 4-hydroxyphenylpyruvate dioxygenase enzyme. With only a few cases ever reported, the symptoms include cognitive and neurological disabilities [22,23].

Interestingly, as previously published, antibodies raised against Phe assemblies was proven to be valuable diagnostic tool in the case of phenylketonuria (PKU), a common autosomal recessive disorder caused by the genetic malfunction of the phenylalanine hydroxylase enzyme that converts Phe to Tyr, resulting in the accumulation of Phe [14,24,25,26]. Importantly, these anti-Phe fibrils antibodies allowed the identification of Phe assemblies in the sera of PKU model mice and in the brains of PKU human patients’ postmortem [11]. Here, to establish a similar diagnostic tool for tyrosinemia, we report the production of specific antibodies against Tyr assemblies and the utilization of these antibodies for immunodetection of Tyr structures in both in vitro and cell culture systems. Moreover, the antibodies could identify Tyr only in the assembled state, since the use of inhibitors that hinder the Tyr self-assembly process also prohibited immunodetection. Furthermore, the antibodies could be used for immunostaining of cultured cells treated with Tyr assemblies. Finally, pre-incubation of the Tyr fibrils with the specific antibodies led to depletion of the structures cytotoxicity. Taken together, this work provides new tools for identification, characterization and understating of the immunological properties of Tyr amyloid-like assemblies and their role in the pathology of tyrosinemia.

## 2. Results and Discussion

### 2.1. Tyrosine Self-Assembly, Antibody Production and Characterization

**Figure 1:**
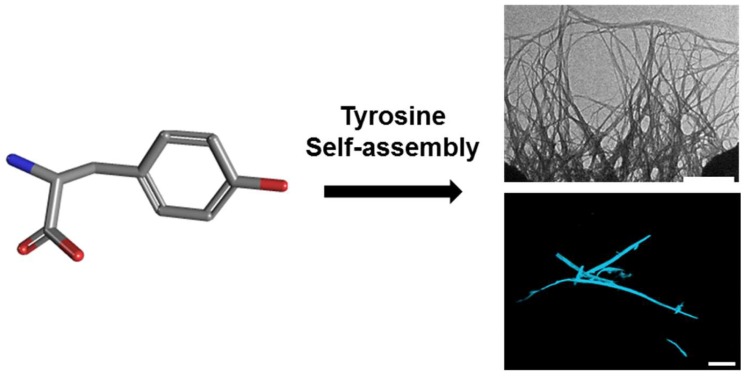
Tyrosine molecular self-assembly. (**Left**) Molecular scheme of monomeric l-Tyr. (**Right**) Transmission electron microscopy (TEM; scale bar: 500 nm; (**top**)) and confocal microscopy following ThT binding (scale bar: 100 µm; (**bottom**)) images of l-Tyr self-assembled ordered supramolecular fibrils.

To generate Tyr amyloid-like fibrils, Tyr (2 mg/mL) was first dissolved in PBS, and the solution was heated to 90 °C to ascertain the monomeric state then gradually cooled to allow the formation of amyloid-like assemblies, as previously described [13]. Tyr assemblies were visualized using TEM and tested ability to bind ThT, an amyloid-specific fluorescent dye that binds to β-sheet rich structures [10] (Figure 1). Moreover, the Tyr assemblies presented the fluorescent signal only when ThT was used, which further verifies their amyloid nature (Supplementary Figure 1). Both mass spectrometry and UV-Vis analysis demonstrated the assemblies’ building block to be Tyr (Supplementary Figure 2).

**Figure 2:**
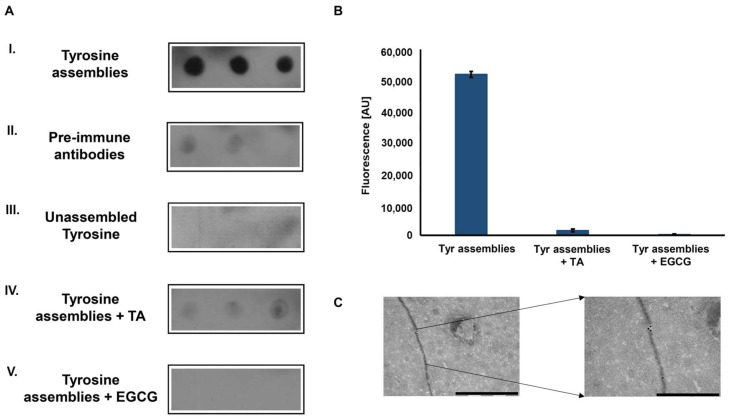
Immunodetection of tyrosine fibrillary assemblies by anti-Tyr antibodies. (**A**) Dot blot assay. I. Blotting of Tyr (2 mg/mL) amyloid-like assemblies probed with anti-Tyr antibodies. II. Blotting of Tyr (2 mg/mL) amyloid-like assemblies probed by antibodies purified from pre-immune rabbits. III. Blotting of unassembled Tyr (0.1 mg/mL) probed by anti-Tyr antibodies. IV-V. Blotting of Tyr (2 mg/mL) structures that were pre-incubated with 0.1 mM TA (IV) or 1 mM EGCG (V) probed by anti-Tyr antibodies (**B**) ThT endpoint fluorescence assay of Tyr (2 mg/mL) structures, pre-incubated in the absence or presence of TA (0.1 mM) and EGCG (1 mM) (20 µM ThT, excitation at 450 nm and emission at 480 nm). (**C**) Left: TEM micrograph of Tyr (2 mg/mL) amyloid-like fibril visualized using anti-Tyr antibodies and a secondary antibody conjugated to 18-nm gold particles, scale bar: 1 µm. Right: magnification of a gold labeled area in the fibril, scale bar: 500 nm.

To examine whether the Tyr amyloid-like assemblies are immunogenic, polyclonal antibodies against these structures were generated. Tyr fibrils solution (4 mg/mL, as previously described [13]) served as an antigen for several immunization cycles in rabbits. Next, the blood serum was collected, and polyclonal IgG antibodies were purified using Protein G column chromatography and used as primary antibodies. The reactivity of the purified polyclonal antibodies was examined using a dot blot immunoassay. The recognition of in vitro assembled Tyr amyloid-like fibrils by the polyclonal antibodies was demonstrated (Figure 2A-I), while antibodies purified from pre-immune rabbits, serving as a negative control, did not recognize the Tyr fibrils (Figure 2A-II). To verify that the antibodies specifically recognize Tyr in the assembled form, a low concentration of Tyr (0.1 mg/mL), in which the amino acid is in its unassembled form (Supplementary Figure 3) was also examined, yielding no reaction (Figure 2A-III).

Tannic acid (TA) and epigallocatechin gallate (EGCG), are two polyphenolic compounds previously shown to inhibit the self-assembly of proteinaceous amyloids [27,28] and recently demonstrated, using time-dependent ThT fluorescence assay, to hinder the Tyr self-assembly process [29]. Pre-treatment of the Tyr solution with either of the polyphenols hindered immune-recognition (Figure 2A-IV–V). Consistently, the inhibition of Tyr assembly by both compounds was verified using a ThT endpoint fluorescence assay, which resulted in a low ThT fluorescence signal, indicating the absence of Tyr amyloid-like fibrils (Figure 2B). To further demonstrate the ability of the anti-Tyr antibodies to specifically bind and recognize Tyr fibrils, an immuno-gold assay was performed. As shown in the TEM images, the fibrils, at a diameter correlating to previous findings [13], were specifically marked with gold-labeled secondary antibodies bound to the anti-Tyr antibodies (Figure 2C).

Altogether, the specific identification of in vitro formed Tyr fibrils by the newly generated anti-Tyr antibodies indicates these assemblies to be immunogenic.

### 2.2. Cellular Internalization and Cytotoxicity of Tyr Assemblies

**Figure 3:**
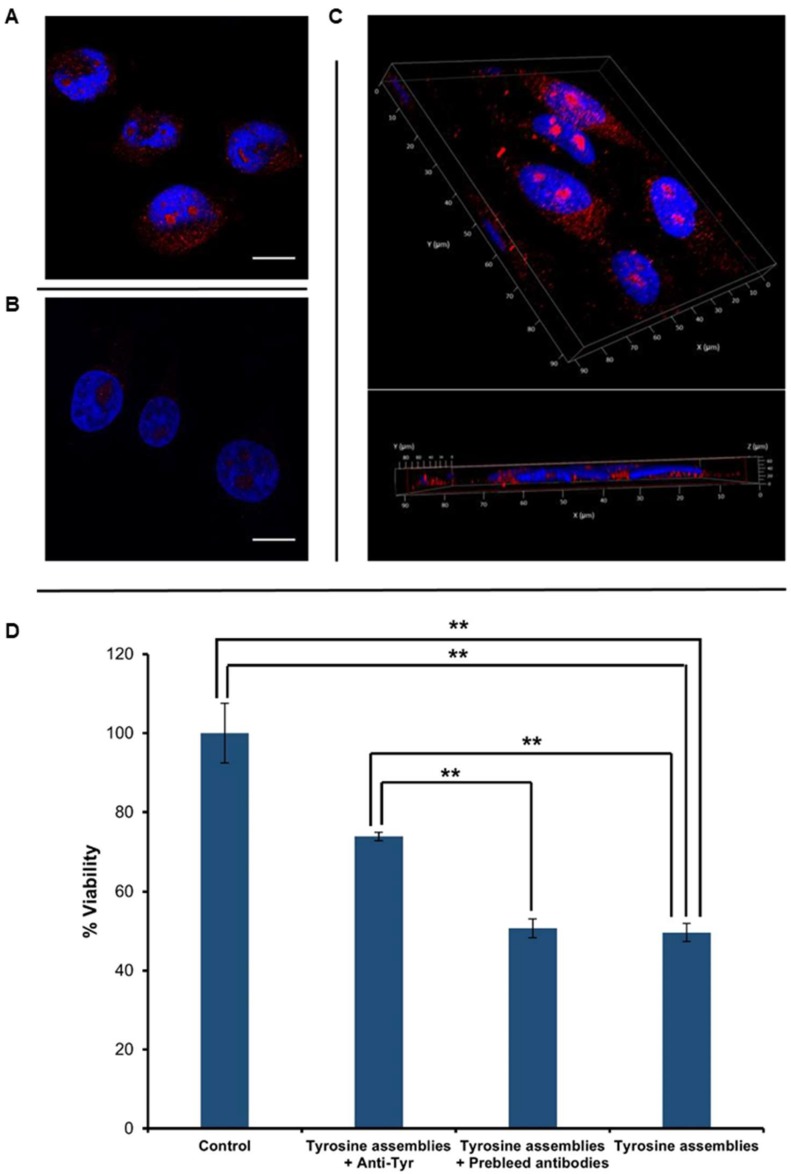
Cellular internalization and toxicity of Tyr assemblies. (**A**–**C**) Tyr was dissolved at 90 °C in cell culture medium, followed by gradual cooling of the solution. The control reflects medium with no Tyr assemblies, which was treated in the same manner. The cells were stained with anti-Tyr antibodies and visualized using confocal microscopy. DAPI (blue) and anti-Tyr staining (red) are shown. Scale bars: 15 μm. (**A**) SH-SY5Y cells treated with medium containing Tyr assemblies. (**B**) Control untreated SH-SY5Y cells. (**C**) 3D volume reconstruction of the Z-series with XZ-slice projection of treated cells. The interval between individual Z-stack serial images was 0.5 μm. (**D**) SH-SY5Y cells were treated with medium containing Tyr fibrils, which were pre-incubated with anti-Tyr antibodies (Tyrosine assemblies + anti-Tyr), Tyr fibrils pre-incubated with pre-immune antibodies (Tyrosine assemblies + pre-immune antibodies), and with medium containing non-treated Tyr fibrils (Tyrosine assemblies), following the addition of the MTT reagent. Absorbance was determined at 570 nm and 680 nm. The results represent three biological repeats. Values are means ± SD, student’s *t*-test, ** *p* < 0.001.

After verifying the reactivity of the anti-Tyr antibodies and their specific recognition of Tyr fibrils in vitro, we aimed to examine the immuno-detection of Tyr structures in cell culture systems. SH-SY5Y neuroblastoma cells were treated with Tyr assemblies for 4 h (Figure 3A), having untreated cells serving as a negative control (Figure 3B). Immunostaining using the anti-Tyr antibodies showed the presence of Tyr assemblies in the treated cells, but not in the control. Moreover, Z-stack analysis and 3D reconstruction clearly demonstrated the internalization of the Tyr assemblies into the treated cells (Figure 3C).

Tyr assemblies (4 mg/mL) were recently shown to trigger a cytotoxic effect, resulting in a 50% decrease in SH-SY5Y cell viability upon 6 h treatment [13]. We were therefore interested in examining the effect of anti-Tyr antibodies on the cytotoxicity triggered by the Tyr assemblies. Utilizing an MTT cell proliferation assay, we found that treatment of SH-SY5Y cells with Tyr assemblies (2 mg/mL) that were pre-incubated overnight with anti-Tyr antibodies resulted in almost 80% cell viability, as compared to 50% following treatment with Tyr amyloid-like assemblies pre-incubated with pre-immune antibodies or with non-treated Tyr assemblies (Figure 3D). Comparably, the use of Tyr assemblies pre-incubated with either EGCG or TA also resulted in a ~80% cell viability [29]. The depletion of Tyr fibrils cytotoxicity triggered by the anti-Tyr antibodies, to a similar extent as the previously studied polyphenolic inhibitors, further indicates their specific recognition of the toxic Tyr species.

## 3. Materials and Methods

### 3.1. Materials

l-Tyrosine (≥99% purity), tannic acid (ACS grade), epigallocatechin gallate (EGCG) (≥95% purity), thiazolyl blue tetrazolium bromide (MTT reagent), 98% Thioflavin T (ThT) and Triton X-100 were purchased from Sigma-Aldrich (Rehovot, Israel). Hoechst 33342 solution was purchased from Thermo-Fisher scientific (Jerusalem, Israel). Dimethylformamide (DMF) was purchased from Bio-lab (Jerusalem, Israel). Sodium n-dodecyl sulfate (SDS) 99% was purchased from Tzamal D-Chem (Petah Tikva, Israel).

#### 3.1.1. Anti-Tyrosine Antibodies Production

Assemblies of Tyr were formed in vitro (see below) and served as antigens in a series of three immunization cycles. Polyclonal IgG antibodies were purified via protein G column and supplied by Abcore Llc (Ramona, CA, USA).

#### 3.1.2. Secondary Antibodies

Monoclonal HRP-conjugated goat anti-rabbit was purchased from Genscript (Piscataway, NJ, USA). Monoclonal 18nm colloidal gold-AffiniPure goat anti-rabbit IgG and polyclonal Cy3-conjugated goat anti-rabbit were purchased from Jackson ImmunoResearch (West Grove, PA, USA).

### 3.2. Experimental Methods

#### 3.2.1. Tyrosine Fibril Formation

Fresh stock of solutions was prepared by dissolving Tyr in either PBS or Dulbecco’s modified Eagle’s medium (DMEM)/Nutrient Mixture F12 (Ham’s) (1:1) at 90 °C, to final concentrations of 0.1 mg/mL for unassembled Tyr and 2 mg/mL for Tyr fibrils, followed by overnight gradual cooling of the solution. To form the inhibited assemblies, Tyr was dissolved at 90 °C in PBS to a final concentration of 2 mg/mL. The inhibitors, EGCG (1 mM) or TA (0.1 mM), were added, followed by overnight gradual cooling of the solution. 

#### 3.2.2. Dot-Blot Assay

Tyr was freshly dissolved at 2 mg/mL and gradually cooled to form amyloid-like assemblies. 50 µL of tyrosine assemblies’ solution was loaded in triplicates onto a PVDF membrane (GE—healthcare), fixed in a Bio-rad dot-blot vacuum manifold and absorbed overnight at 4 °C. The membrane was blocked with 3% BSA (Amresco) for 2 h. Primary antibody, diluted 1:1000, was added for another 2 h. Secondary antibody was diluted 1:15,000 and used for 1 h. Between these stages, 3 TBS-T 0.1% washes, 5 min each, were performed. Blots were developed using Luminata Forte Western HRP substrate and visualized on an X-ray film.

#### 3.2.3. ThT Endpoint Fluorescence assay

Tyr was dissolved at 90 °C in PBS to a final concentration of 2 mg/mL, to obtain monomeric solutions of the metabolite. Next, the solutions were mixed with the inhibitors, EGCG (1 mM) and TA (0.1 mM), in a black 96-well, clear and flat bottom microplate (Greiner bio-one, Kremsmünster, Austria). As a control, Tyr was diluted with PBS alone to the same final concentration. ThT reagent in PBS was added to a final concentration of 20 µM. Following excitation at 450 nm, ThT emission data at 480 nm was measured and recorded using a TECAN Infinite® 200 PRO plate reader.

#### 3.2.4. Rabbit Antibody Immuno-Gold Assay

A 10-μL aliquot of 2 mg/mL Tyr assemblies’ solution was placed on 400-mesh copper grids. After 2 min, excess fluids were removed, and the grid was allowed to dry for 2 min. Then, the grid was blocked with 1% (*w*/*v*) BSA and 3% (*w*/*v*) goat serum for 30 min. Samples were incubated with the rabbit immuno-serum diluted 1:200 in 1% milk in PBS for 30 min, washed five times with 0.1% BSA, and then incubated with goat anti-rabbit conjugated with 18-nm gold (Jackson ImmunoResearch; cat no. 111-215-144; 1:20) for 30 min and similarly washed. Samples were viewed using a JEM-1400Plus electron microscope operating at 80 kV.

#### 3.2.5. Immuno-Staining

Cells were grown to 50% confluence on poly-l-lysine coated cover-slips with (Mercury) in 24-well plates. The cells were then treated with cell growth medium containing Tyr (4 mg/mL) amyloid-like assemblies. The control cells were treated with medium with no Tyr that was processed in the same manner. The cells were then rinsed with PBS and fixed in 4% PFA (Bar Naor) for 15 min at room temperature. The cells were washed twice with ice-cold PBS and treated with 0.25% Triton X-100 for 10 min at room temperature to allow cellular permeabilization. After thoroughly washing the cells, blocking was performed using 1% BSA (Amresco) overnight at 4 °C. Then, the cells were stained using a rabbit polyclonal anti-Tyr antibody diluted 1:100 in blocking solution, for 1 h at room temperature. Slides were washed three times with PBS and anti-rabbit Cy3-conjugated secondary antibody diluted 1:200 in blocking solution was added for another 30 min at room temperature in the dark. Finally, cells were washed three times and the cover slips were mounted using 15 µL Vectashield Antifade Mounting Medium with DAPI (Vector laboratories). Imaging was performed using SP8 inverted confocal microscopy (Leica Microsystems, Wetzlar, Germany). Excitation/emission wavelengths were 412/450 nm for DAPI and 548/561 nm for Cy3.

#### 3.2.6. Cytotoxicity Analysis

SH-SY5Y cells (2 × 10^5^ cells/mL) were cultured in 96-well tissue microplates (100 µL per well) and allowed to adhere overnight at 37 °C. Tyr assemblies were formed in DMEM/Nutrient Mixture F12 (Ham’s) (1:1) (Biological Industries) at a concentration of 2 mg/mL, as outlined above. Only half of each plate was seeded with cells, with the other half used as a blank control. Medium with no Tyr, which was treated in the same manner, served as a negative control, represented by zero. Medium (100 µL) with or without assemblies was added to each well. After overnight incubation at 37 °C, cell viability was evaluated using 3-(4,5-dimethylthiazolyl-2)-2, 5-diphenyltetrazolium bromide MTT cell proliferation assay (Sigma-Aldrich, Rehovot, Israel) according to the manufacturer’s instructions. Briefly, after overnight incubation at 37 °C with the metabolite, 10 µL of 5 mg/mL MTT reagent dissolved in PBS was added to each of the 96 wells, followed by 4 h of incubation at 37 °C. Next, 100 µL extraction buffer (50%DMF, 20%SDS in DDW) was added to the wells, followed by 30 min incubation at 37 °C in the dark. Finally, color intensity was measured using an ELISA plate reader at 570 nm and background subtraction at 680 nm. The results represent three biological repeats. Values are means ± SD, student’s *t*-test, ** *p* < 0.001.

## 4. Conclusions

In the current work, the production of anti-Tyr antibodies raised against Tyr amyloid-like assemblies is presented. The identification of in vitro Tyr fibrils by these antibodies indicates the immunogenic properties of the assemblies, as previously shown for protein and polypeptide amyloid assemblies [30,31,32,33,34]. Moreover, antibodies purified from the pre-immune sera could not detect the Tyr assemblies, further supporting the characterization of Tyr assemblies as immunological entities. The specificity and distinct recognition of the Tyr amyloid-like assemblies was verified by the lack of antibodies recognition using low concentration (unassembled) Tyr and Tyr self-assembled samples pre-treated with polyphenol inhibitors. In addition, we demonstrated the detection of internalized Tyr assemblies by the anti-Tyr antibodies in a cell culture model. Finally, the depletion of Tyr assemblies’ cytotoxicity by the anti-Tyr antibodies was established, possibly further indicating the pathological role of the Tyr amyloid-like structures.

The production of anti-Tyr antibodies reflects an additional evidence for the extension of the “amyloid hypothesis” to include various metabolites. The generated anti-Tyr antibodies provide a valuable tool, offering new methodologies to decipher the role of Tyr fibrils in tyrosinemia pathology, as well as the Tyr self-assembly mechanism. Recently the Tyr assemblies were shown to interact and penetrate model membranes, as known for protein and polypeptide amyloids, possibly indicating their course of apoptotic triggering and cell internalization [35]. Thus, these antibodies may also provide a possible future therapeutic direction via utilization of the anti-Tyr antibodies, which may inhibit the formation of the Tyr toxic species. We hope that the observations presented here will promote further investigation and usage of the anti-Tyr antibodies in the context of other amyloid structures.

## Supplementary Materials

The supplementary materials are available online.
