# Supplementary material for: Antibodies towards Tyrosine Amyloid-Like Fibrils Allow Toxicity Modulation and Cellular Imaging of the Assemblies
Source: Molecules. 2018 May 26;23(6):1273. doi: 10.3390/molecules23061273 (PMC6100058; doi:10.3390/molecules23061273)
Supplement: Supplementary file 1 [file molecules-23-01273-s001.zip › molecules-293691-SI.pdf]

Supplementary materials

This file includes:

- **Supplementary figure 1**. Confocal imaging of Tyr assemblies (2 mg/ml).
- **Supplementary figure 2.** Identification of Tyr assemblies' building block.
- **Supplementary figure 3.** Unassembled Tyr solution.
- **Supplementary methods**

Supplementary results

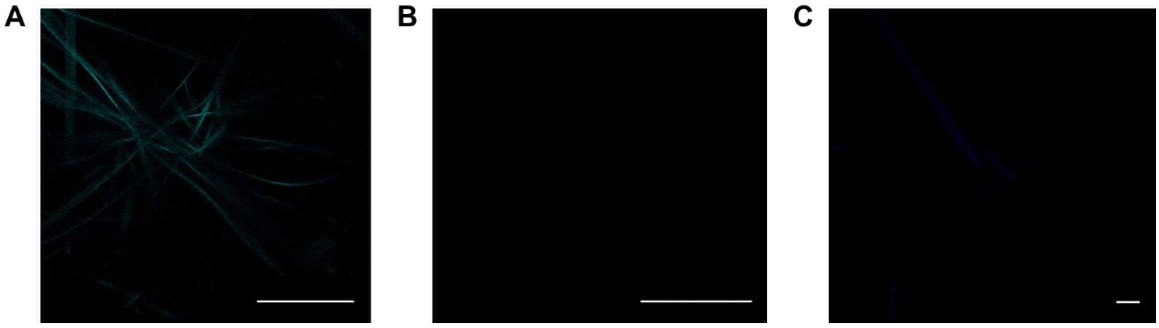

Supplementary figure 4. Confocal imaging of Tyr assemblies (2 mg/ml). (**A**) Tyr assemblies stained with ThT dye. (**B**) Tyr assemblies without ThT dye. (**C**) Tyr assemblies stained with Hoechst 33342 dye. Scale bars: 20 μm

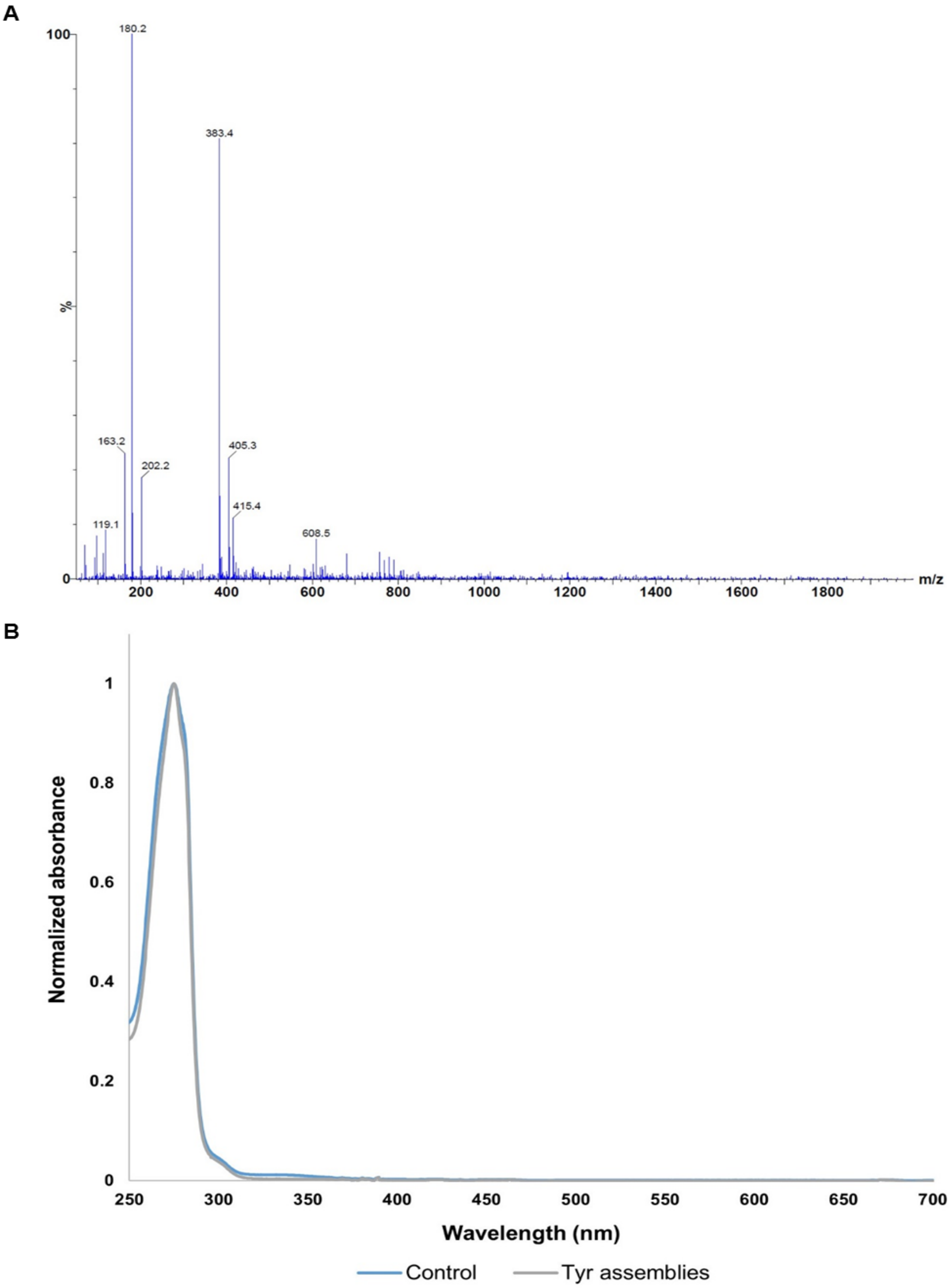

Supplementary figure 5. Identification of Tyr assemblies' building block. (**A**) Mass spectroscopy of Tyr assemblies. Dominant mass peaks: 180.2 – Tyr, 384.3 – Tyr dimer with sodium. Minor mass peaks: 163.2 – Tyr without amine group, 202.2 – Tyr with sodium, 405.3 – Tyr dimer with two sodium ions. (**B**) UV-Vis spectra of Tyr assemblies. Solubilized Tyr assemblies (grey) and unassembled Tyr used as a control (blue) are shown.

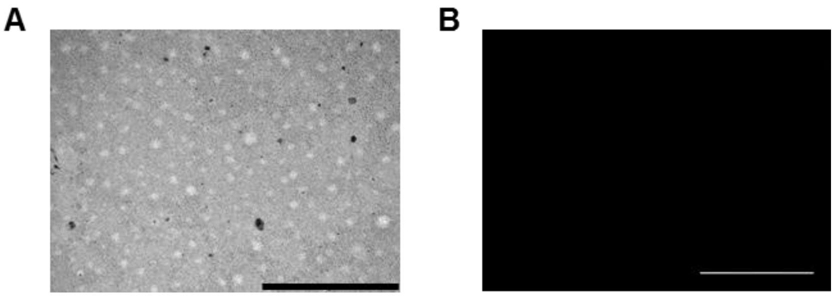

Supplementary figure 6. Unassembled Tyr solution. Tyr at a concentration of 0.1 mg/ml was heated to 90° C and cooled down gradually. (**A**) TEM micrograph, scale bar: 1μm. (**B**) Staining with ThT dye as visualized by confocal microscopy, scale bar: 20μm.

Supplementary methods

ThT binding assay

Tyr was dissolved at 90°C in PBS at a concentration of 4 mg/ml, followed by gradual cooling of the solution. ThT solution (10 μl, 4 mM, PBS x1) was mixed with 10 μl of the Tyr solution to a final concentration of 2 mg/ml and placed on a glass microscope slide. The stained samples were visualized using SP8 inverted confocal microscope (Leica Microsystems, Wetzlar, Germany), with excitation and emission wavelengths of 458 and 485 nm, respectively.

Mass spectroscopy

The Tyr assemblies (2 mg/ml) sample was prepared for mass spectrometry by a 20-fold dilution in water to a final concentration below the solubility limitation of Tyr in water (0.1 mg/ml). Mass spectrometry was recorded using an MALDI-SYNAPT mass spectrometer (Waters, USA).

UV-Vis spectra

For UV-Vis measurements, 1 mL of Tyr dissolved assemblies (0.1 mg/ml, as described in the mass spectroscopy section) or soluble Tyr at the same concentration (serving as the control) were added to a rectangular quartz cuvette with an optical path-length of 1 cm (Starna). UV-Vis spectra in the range of 250-700 nm were acquired using a T60 spectrophotometer (PG Instruments) at a spectral resolution of 0.2 nm and corrected using PBS as a baseline.
